# The effect of oral butyrate on colonic short-chain fatty acid transporters and receptors depends on microbial status

**DOI:** 10.3389/fphar.2024.1341333

**Published:** 2024-03-26

**Authors:** Karla Vagnerová, Tomáš Hudcovic, Martin Vodička, Peter Ergang, Petra Klusoňová, Petra Petr Hermanová, Dagmar Šrůtková, Jiří Pácha

**Affiliations:** ^1^ Institute of Physiology, Czech Academy of Sciences, Prague, Czechia; ^2^ Institute of Microbiology, Czech Academy of Sciences, Nový Hrádek, Czechia; ^3^ Department of Physiology, Faculty of Science, Charles University, Prague, Czechia

**Keywords:** dextran sulfate, colitis, butyrate, germ-free (GF), short chain fatty acid (SCFA), butyrate transporters, butyrate receptors, microbiota

## Abstract

Butyrate, a metabolite produced by gut bacteria, has demonstrated beneficial effects in the colon and has been used to treat inflammatory bowel diseases. However, the mechanism by which butyrate operates remains incompletely understood. Given that oral butyrate can exert either a direct impact on the gut mucosa or an indirect influence through its interaction with the gut microbiome, this study aimed to investigate three key aspects: (1) whether oral intake of butyrate modulates the expression of genes encoding short-chain fatty acid (SCFA) transporters (*Slc16a1*, *Slc16a3*, *Slc16a4*, *Slc5a8*, *Abcg2*) and receptors (*Hcar2*, *Ffar2*, *Ffar3*, *Olfr78*, *Olfr558*) in the colon, (2) the potential involvement of gut microbiota in this modulation, and (3) the impact of oral butyrate on the expression of colonic SCFA transporters and receptors during colonic inflammation. Specific pathogen-free (SPF) and germ-free (GF) mice with or without DSS-induced inflammation were provided with either water or a 0.5% sodium butyrate solution. The findings revealed that butyrate decreased the expression of *Slc16a1*, *Slc5a8*, and *Hcar2* in SPF but not in GF mice, while it increased the expression of *Slc16a3* in GF and the efflux pump *Abcg2* in both GF and SPF animals. Moreover, the presence of microbiota was associated with the upregulation of *Hcar2*, *Ffar2,* and *Ffar3* expression and the downregulation of *Slc16a3*. Interestingly, the challenge with DSS did not alter the expression of SCFA transporters, regardless of the presence or absence of microbiota, and the effect of butyrate on the transporter expression in SPF mice remained unaffected by DSS. The expression of SCFA receptors was only partially affected by DSS. Our results indicate that (1) consuming a relatively low concentration of butyrate can influence the expression of colonic SCFA transporters and receptors, with their expression being modulated by the gut microbiota, (2) the effect of butyrate does not appear to result from direct substrate-induced regulation but rather reflects an indirect effect associated with the gut microbiome, and (3) acute colon inflammation does not lead to significant changes in the transcriptional regulation of most SCFA transporters and receptors, with the effect of butyrate in the inflamed colon remaining intact.

## Introduction

Inflammatory bowel disease (IBD) including its major clinical forms ulcerative colitis (UC) and Crohn’s disease (CD), is a chronic, recurrent disease characterized by intestinal inflammation whose etiology is still poorly understood. Nevertheless, the pathogenesis of IBD is multifactorial and results from genetic predisposition, immunological status, and environmental factors, including intestinal microbiota disbalance (Graham and Xavier, 2020). Gut microbial dysbiosis was detected both in patients with UC and CD and animal models of IBD (Ni et al., 2017; Facchin et al., 2020) and was associated with a reduced number of short-chain fatty acid (SCFA)-producing bacteria and reduced butyrate concentration (Machiels et al., 2014; Laserna-Mendieta et al., 2018).

SCFAs including butyrate, propionate, and acetate, are produced by the fermentation of nondigestible carbohydrates (dietary fibers) by the gut microbiota and play an important role in maintaining intestinal barrier functions and immune homeostasis. SCFAs, particularly butyrate, have been shown to regulate the proliferation and differentiation of intestinal epithelial cells and their expression of mucins, antimicrobial peptides, and tight junctions proteins; furthermore, butyrate exerts anti-inflammatory effects in immune cells (Gonçalves et al., 2018; Recharla et al., 2023). SCFAs interact with target epithelial or immune cells either via cell-surface G-protein-coupled receptors or by entering cells and controlling gene expression by the direct inhibition of histone deacetylases (Dalile et al., 2019). Previous studies have established that butyrate and other SCFAs can enter enterocytes via passive nonionic diffusion or carrier-mediated transport, facilitating the transport of SCFAs derived from gut microbiota. This transport is mediated by the electroneutral H^+^-dependent monocarboxylate transporters MCT1, MCT4, and MCT5 and the electrogenic, Na^+^-dependent monocarboxylate transporter SMCT1, which are encoded by the *Slc16a1*, *Slc16a3*, *Slc16a4,* and *Slc5a8* genes, respectively (Gill et al., 2005; Cresci et al., 2010; Al-Mosauwi et al., 2016). Moreover, the efflux of butyrate has been demonstrated through the ABCG2 efflux pump, expressed in the apical membrane of intestinal epithelial cells (Gonçalves et al., 2011). In addition to their interactions with transporters, SCFAs act as ligands capable of activating cell signaling pathways in enterocytes via at least five distinct membrane receptors: GPR109A/HCAR2 (hydrocarboxylic acid receptor 2), FFAR2 and FFAR3 (free fatty acid receptor 2 and 3), OLFR78 (olfactory receptor 78), and OLFR558/OR51E1 (olfactory receptor 558) (Priori et al., 2015; Priyadarshini et al., 2018; Halperin Kuhns et al., 2019; Nishida et al., 2021).

The findings that microbiota-derived butyrate plays an important role in maintaining intestinal barrier integrity, gut homeostasis, and reducing gut inflammation (Gonçalves et al., 2018) led to many studies investigating the therapeutic implications of butyrate treatment for IBD (Recharla et al., 2023). In animal models of colitis (DSS-, TNBS- or IL10^−/−^-colitis), oral butyrate supplementation attenuated the disease activity index, inflammation and mucosal lesions (Vieira et al., 2012; Ji et al., 2016; Lee et al., 2017; Chen et al., 2018), although some studies failed to show benefits following butyrate treatment (Lee et al., 2022). Moreover, butyrate failed to protect against TNBS-colitis in *Hcar2* ^*−/−*^ mice (Chen et al., 2018), and *Ffar2* ^*−/−*^ and *Ffar3* ^*−/−*^ mice were found to be more susceptible to TNBS-colitis (Kim et al., 2013). Similarly, the beneficial effect of dietary fibers in DSS colitis was diminished in *Hcar2* ^*−/−*^, *Ffar2* ^*−/−*^, and *Slc5a8* ^*−/−*^ mice (Gurav et al., 2015; Macia et al., 2015). However, the mechanisms through which oral butyrate operates remain incompletely understood, particularly whether it can directly affect the gut mucosa or if its effects are mediated indirectly through its influence on the gut microbial community (Dou et al., 2020; Facchin et al., 2020; Lee et al., 2022). Therefore, this study aimed to compare the effects of butyrate on SCFA receptors and transporters in the presence and absence of gut microbiota and intestinal inflammation.

## Materials and methods

### Animals, treatments, and sample preparation

The experiments were performed on 37 two-month-old specific pathogen-free (SPF) and 34 germ-free (GF) female BALB/c mice (Institute of Microbiology, Nový Hrádek, Czech Republic), which were maintained on a 12 h/12 h light/dark cycle and had free access to autoclaved tap water and an irradiated sterile pellet diet of Altromin 1414 (Altromin, Lage, Germany). The GF mice were kept under sterile conditions in Trexler-type isolators since birth. The sterility was monitored routinely by the aerobic and anaerobic cultivation of mouse feces and swabs from the isolator. Colitis was induced by replacing drinking water with 2.5% dextran sulfate sodium (DSS, M.W. 36–50 kDa; MP Biomedicals, Illkirch, France) in water for 7 days (Hudcovic et al., 2001). Sodium butyrate was administered in drinking water at a concentration of 0.5% (Vieira et al., 2012; Chen et al., 2018).

**FIGURE 1:**
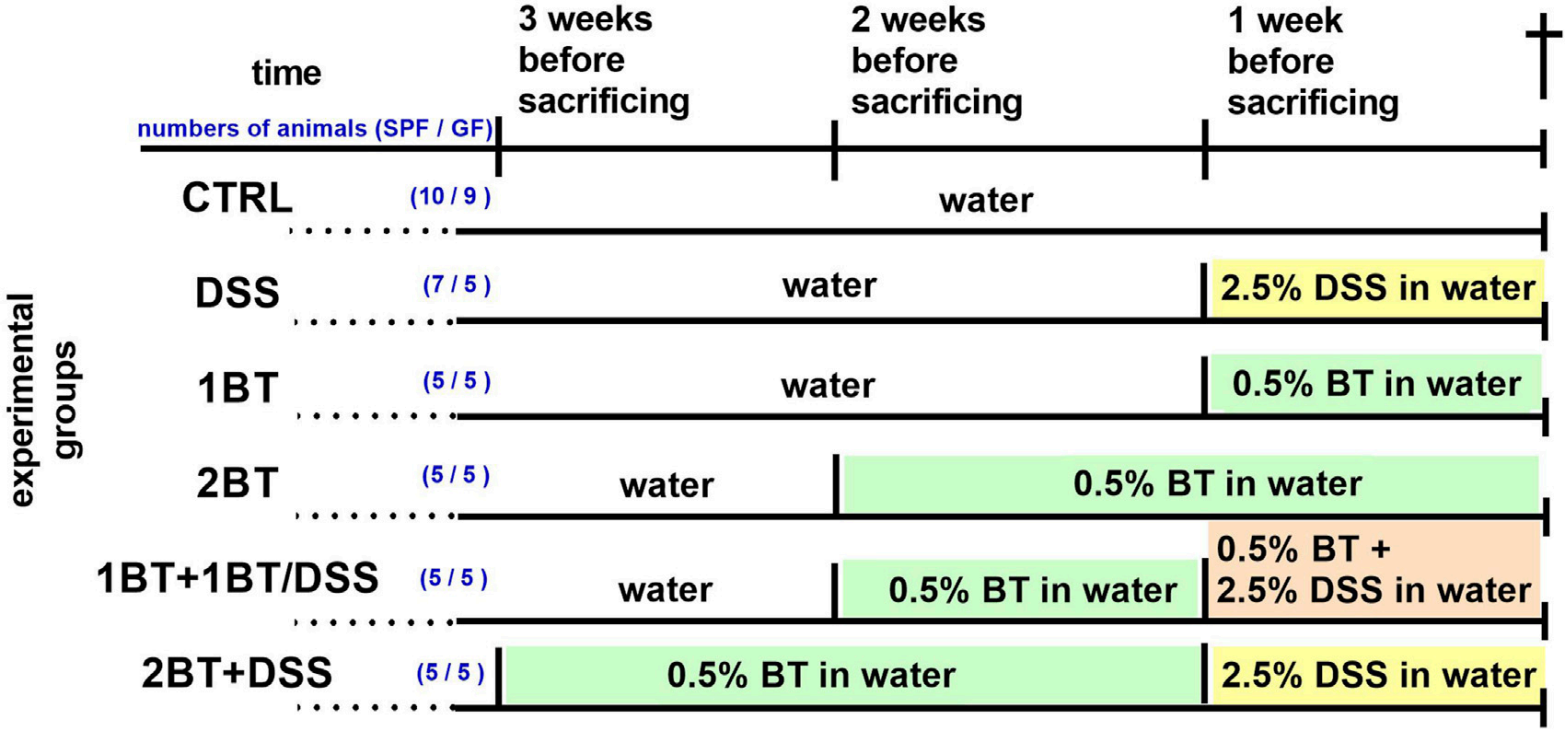
Experimental timeline. DSS, dextran sodium sulfate; BT, sodium butyrate; for more details see the text. The number of animals in each experimental group is given in parentheses (SPF/GF mice).

Both SPF and GF mice were divided into the following six groups (Figure 1): (1) the control group (CTRL), given water without DSS and sodium butyrate; (2) the DSS group, given 2.5% DSS solution in water for 1 week; (3) the butyrate group, given 0.5% sodium butyrate solution in water for 1 week (1BT); (4) the butyrate group, given 0.5% sodium butyrate solution in water for 2 weeks (2BT); (5) the butyrate and DSS group (1BT+1BT/DSS), given sodium butyrate (0.5%) solution 1 week followed by a mixture of sodium butyrate (0.5%) and DSS (2.5%) the following week; and (6) the butyrate and DSS group (2BT + DSS), in which mice received 0.5% sodium butyrate for 2 weeks and 2.5% DSS solution the following week. All mice were anesthetized with isoflurane vapor and decapitated, and the colonic tissue was collected, flash-frozen in liquid nitrogen, and stored at −80°C. The present study builds upon previous research conducted by Jourova et al. (2022) and Satka et al. (2022). Their work demonstrated that administering butyrate to SPF mice for 2 weeks before subjecting them to DSS treatment helped reduce symptoms of intestinal inflammation. These symptoms included clinical indicators, such as colon length shortening, histopathological changes, and decreased colonic epithelial leakiness. Conversely, GF mice showed only a minor increase in clinical score and colon length shortening under similar conditions.

The experiments were approved by the Committee for the Protection and Use of Experimental Animals of the Institute of Microbiology, v. v. i, Czech Academy of Sciences (approval ID: 21/2018).

#### Histological assessment

Tissues from the distal colon were washed in phosphate-buffered saline, fixed in Carnoy’s fluid, embedded in paraffin, sectioned (7 μm) and stained with hematoxylin and eosin. The samples were observed using an Olympus BX 40 microscope equipped with an Olympus Camedia DP 70 digital camera, and subsequent image analysis was conducted utilizing Olympus DP-Soft software. The colonic crypt length was measured as described previously (Hausmann et al., 2011). Colonic crypt length was assessed solely in well-oriented crypts where the entire invagination from the colonic surface was distinctly visible. Twenty individual crypt measurements per animal were analyzed from six mice in each group.

#### Sample preparation and gene expression analysis

Total RNA was isolated from 30–50 mg of the frozen distal colon using RNeasy Plus universal Mini Kit (Qiagen, Hilden, Germany) according to the manufacturer’s instructions. Due to DSS contamination of samples and inhibition of the subsequent reactions, RNA samples were purified using the GenElute Mammalian Total RNA miniprep kit (Sigma-Aldrich, Merck KGaA, Darmstadt, Germany) and DNase treatment using the On-Column DNase I Digestion set (Sigma-Aldrich). Isolated total RNA was transcribed to cDNA using random hexamers and the HighCapacity cDNA Reverse Transcription Kit (Life Technologies, Carlsbad, Ca, USA). Quantitative polymerase chain reactions were performed in the LightCycler 480 PCR System (Roche Diagnostic GmbH, Mannheim, Germany) using 5x Hot FIREpol Probe qPCR Mix Plus (ROX) (Solis BioDyne, Tartu, Estonia) and predesigned TaqMan Assays (Life Technologies) for monocarboxylate transporter 1 (*Slc16a1*, Mm01306379_m1), monocarboxylate transporter 4 (*Slc16a3*, Mm00446102_m1), monocarboxylate transporter 5 (*Slc16a4*, Mm00525195_m1), sodium-coupled monocarboxylate transporter 1 (*Slc5a8*, Mm00520629_m1), breast cancer resistance protein (*Abcg2*, Mm00496364_m1), hydrocarboxylic acid receptor 2 (*Hcar2*, Mm01199527_m1), free fatty acid receptor 2 (*Ffar2,* Mm02620654_s1), free fatty acid receptor 3 (*Ffar3,* Mm07294891_g1), olfactory receptor 78 (*Olfr 78,* Mm00628116_m1), olfactory receptor 558 (*Olfr558,* Mm01279850_m1), mucin 2 (*Muc2,* Mm01276696_m1), gut hormone peptide YY (*Pyy*, Mm00520715_m1), tumor necrosis factor α (*Tnfα*, Mm00443258_m1), and interleukin 1β (*IL-1*β, Mm00434228_m1). The quantity of the transcripts was determined using the standard curve method with serial 3-fold dilutions of the mixed cDNA sample, and the quantity of the transcripts of the genes of interest was calculated relative to the geometric mean of the reference genes succinate dehydrogenase subunit A (*Sdha*, Mm01352366_m1) and hypoxanthine-guanine phosphoribosyl transferase 1 (*Hprt*, Mm01545399_m1).

### Statistical analysis

All quantitative data were analyzed using GraphPad Prism 8 software (GraphPad, La Jolla, CA, USA) and are presented as the mean ± SEM. Data were assessed for normality (Shapiro-Wilk test) and for variance equality (Brown-Forsythe test). When necessary, data were transformed to fit assumptions of normality and homogeneity of variance before analysis (data in graphs are nontransformed). One-way analysis of variance (ANOVA) followed by Tukey’s *post hoc* test was performed to examine statistical significance in datasets of multiple groups. Two-way ANOVA followed by Tukey’s *post hoc* test was used to analyze the interaction between the effects of butyrate treatment and gut microbial status on the expression of butyrate transporters and receptors in the murine colon. Student’s t-test was performed to examine the statistical significance between two groups. *p* values less than 0.05 were considered statistically significant.

## Results

### The regulation of SCFA transporters and receptors in GF and SPF mice given oral butyrate

**FIGURE 2:**
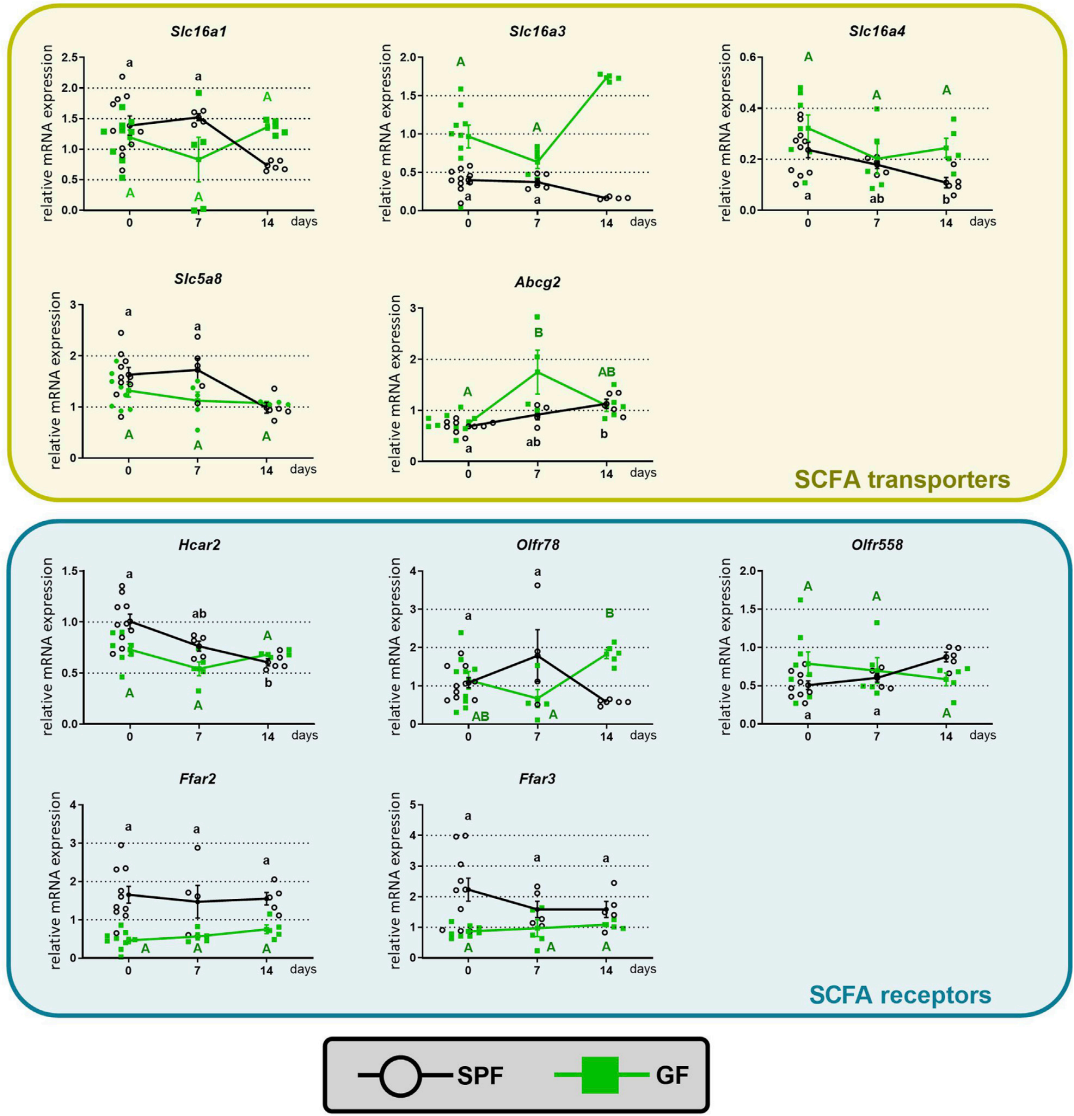
Time course of the effect of butyrate on the expression of colonic SCFA transporters and receptors in specific pathogen-free (SPF) and germ-free (GF) mice. The mice drank either water (CTRL group) or 0.5% sodium butyrate solution for 7 (1BT group) or 14 days (2BT group). The results are presented as the mean ± SEM (*n* = 4–10 per group). The data for SPF (lowercase letters) and GF (uppercase letters) were analyzed separately by one-way ANOVA followed by Tukey’s *post hoc* test. Values with same letters are not statistically different.

Diets containing dietary fibers, the main substrates for bacterial fermentation and the production of SCFAs, stimulate colonic SCFA transport. To test how oral butyrate affects the mRNA abundance of colonic SCFA transporters and receptors, mice received sodium butyrate in drinking water for 1 or 2 weeks (experimental design, Figure 1). Comparison of the groups CTRL, 1BT, and 2BT showed that the responses of GF and SPF mice were different (Figure 2). In SPF mice, butyrate significantly decreased the expression of the solute carriers *Slc16a1*, *Slc16a3*, *Slc16a4*, and *Slc5a8* and increased the expression of the efflux pump *Abcg2* after 2 weeks. The receptors *Hcar2* and *Olfr78* were also significantly decreased, whereas *Olfr558* expression was upregulated and *Ffar2* and *Ffar3* expression was not changed. In contrast, butyrate in GF mice altered *Slc16a3* and *Olfr78* expression, but in the opposite direction compared to that of SPF animals.

**FIGURE 3:**
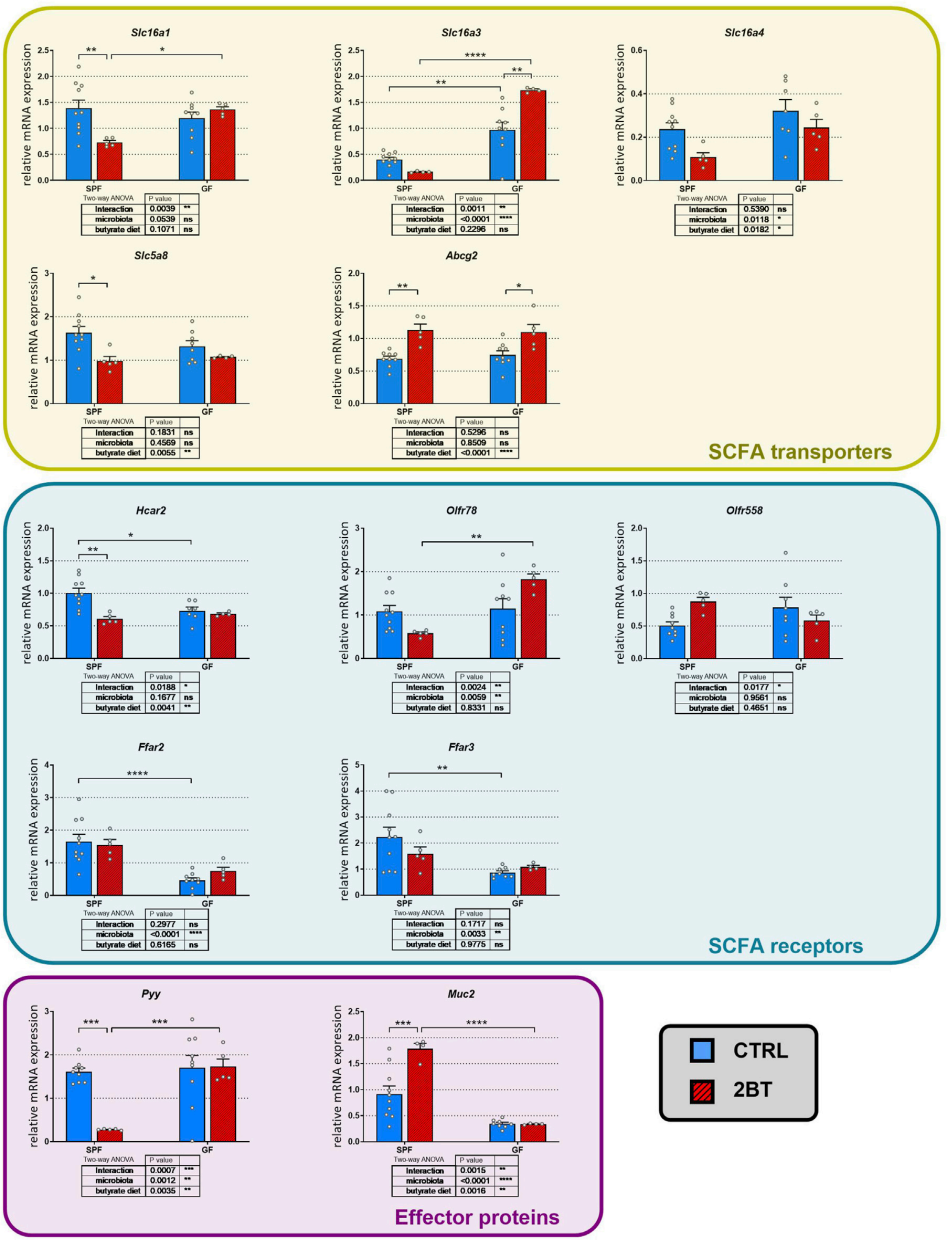
Effects of the microbiota and butyrate on the expression of genes encoding colonic SCFA transporters and receptors, gut hormone PYY, and mucin 2 in specific pathogen-free (SPF) and germ-free (GF) mice. CTRL, mice receiving standard diet and water *ad libitum*; 2BT, mice receiving standard diet and sodium butyrate solution (0.5%) replacing drinking water in the last 14 days before sacrifice. The results are presented as the mean ± SEM (n = 4–10 per group) and were analyzed by a two-way ANOVA followed by Tukey’s *post hoc* test. The results of two-way ANOVA are given in tables and the results of *post hoc* tests in the graphs: *p < 0.05; **p < 0.01; ***p < 0.001, ****p < 0.0001; ns, not significant.

To investigate the influence of the microbiota on the effect of butyrate, we further analyzed the interaction between the gut microbiota and the 2-weeks butyrate treatment on the expression of SCFA transporters and receptors in the distal colon of mice (Figure 3). Two-way ANOVA revealed that microbial status had a significant effect on the expression of the *Slc16a3* and *Slc16a4* transporters and the *Olfr78*, *Ffar2,* and *Ffar3* receptors, and butyrate had a significant effect on the treatment on *Slc16a4*, *Slc5a8* and *Abcg2* transporters and the *Hcar2* receptor. The interaction between butyrate treatment and microbial status significantly impacted the transporters *Slc16a1* and *Slc16a3* and some receptors (*Hcar2*, *Olfr78*, and *Olfr558*).

Interestingly, *post hoc* comparison revealed that butyrate downregulated the expression of *Slc16a1* (*p* < 0.01), *Slc5a8* (*p* < 0.05), and *Hcar2* (*p* < 0.01) in SPF but not GF mice, whereas in the case of *Slc16a4*, the decrease was not significant in SPF mice. In contrast, butyrate treatment upregulated *Slc16a3* expression in GF mice (*p* < 0.01) and *Abcg2* expression in both SPF and GF mice (*p* < 0.05 and 0.01, respectively). The effect of microbiota on the expression of SCFA transporters and receptors differed from that of butyrate. Microbiota downregulated the expression of *Slc16a3* (*p* < 0.01) but had an opposite effect on the expression of *Hcar2* (*p* < 0.05), *Ffar2* (*p* < 0.0001), and *Ffar3* (*p* < 0.01). In addition, the absence of microbiota prevented the decrease in *Slc16a1*, *Slc5a8* and *Hcar2* expression induced by increased butyrate intake.

To investigate whether the interplay between microbial status and oral butyrate can extend its influence on other local processes in the gut, we conducted additional studies to examine the effects of butyrate and microbiota on the expression of *Pyy* and *Muc2* genes. These genes encode the gut hormone PYY and mucin 2, respectively, both of which are known to be modulated by butyrate. Additionally, the secretion of PYY is linked to the activation of FFAR2 receptors, as reviewed recently (Dalile et al., 2019). For both *Pyy* and *Muc2*, two-way ANOVA showed that butyrate treatment, microbial status and the butyrate treatment x microbial status interaction had a significant effect (Figure 3). Butyrate administration significantly downregulated *Pyy* expression (*p* < 0.001) and upregulated *Muc2* expression (*p* < 0.001) in SPF but not GF mice and the GF conditions suppressed expression of *Muc2* just at the level of significance (*p* = 0.0504).

These data suggest that butyrate administration for 2 weeks has an impact on the colonic expression of SCFA transporters and receptors, mucin, and the gastrointestinal hormone PYY and that their expression is modulated by the presence/absence of gut microbiota.

### Effect of butyrate on acute DSS-induced colitis

**FIGURE 4:**
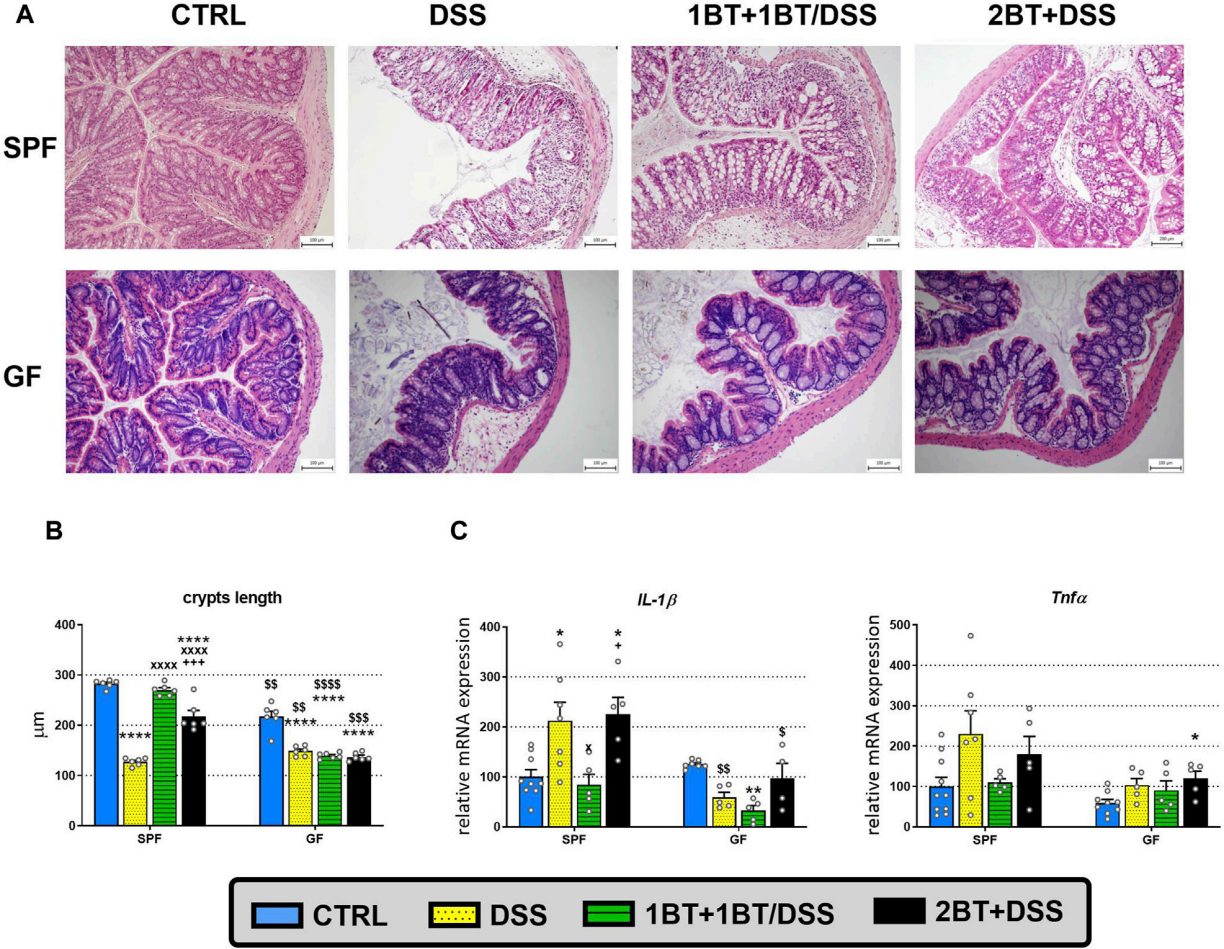
Dextran sodium sulfate (DSS)-induced colitis and the effect of oral butyrate (BT) on crypt length and expression of proinflammatory cytokines. **(A)** Hematoxylin and eosin staining of colon tissue from specific pathogen-free (SPF) and germ-free (GF) mice treated with DSS. The histopathological changes in the colonic mucosa following DSS treatment were assessed through histological scoring at the end of the experiment. These changes are illustrated using representative histological sections. **(B)** The length of colonic crypts and **(C)** mRNA expression of interleukin 1β (*IL-1*β) and tumor necrosis factor α (*Tnfα*) in SPF and GF mice treated with DSS and BT. CTRL, control, untreated mice; DSS, mice that drank 2.5% DSS for 7 days; 1BT+1BT/DSS, mice that drank 0.5% BT solution for 1 week and then the mixture of 0.5% BT and 2.5% DSS for the second week; 2BT + DSS, mice pretreated with 0.5% BT for 2 weeks before drinking 2.5% DSS the following week. The results are presented as the mean ± SEM (*n* = 6 per group for the measurement of crypt lengths; *n* = 4–10 per group for the analysis of proinflammatory cytokine expression) and were analyzed by one-way ANOVA separately for SPF and GF mice. Different from the CTRL group: *p < 0.05, **p < 0.01, ****p < 0.0001; different from the DSS group: ^x^ *P* < 0.05, ^xxxx^ *P* < 0.0001; different from the 1BT+1BT/DSS group: ^+^ *p* < 0.05, ^+++^ *p* < 0.001. Differences between SPF and GF mice in identical experimental groups were compared using Student’s t-test: ^$^ *p* < 0.05, ^$$^ *p* < 0.01, ^$$$^ *p* < 0.001, ^$$$$^ *p* < 0.0001.

As SCFAs exert anti-inflammatory effects in the intestinal mucosa, we investigated the expression of SCFA transporters and receptors during inflammation in mice with acute DSS-induced colitis. First, we studied the manifestation of the disease at the level of colonic crypt length shortening and inflammation (Figure 4). The histological examination (Figure 4A) of SPF mice revealed significant inflammatory cell infiltration into the lamina propria, thickening of the submucosa, loss of the epithelial layer, and disappearance of mucosal crypts in the colonic wall of DSS-treated controls (group DSS; grade 3.3 ± 0.3). These changes were also observed to a lesser extent in mice pretreated with butyrate for 1 week and cotreated with butyrate and DSS the following week (group 1BT+ 1BT/DSS; grade 2.2 ± 0.2). In contrast, mice pretreated with butyrate for 2 weeks displayed a notable inhibitory effect on DSS-induced histological changes (group 2BT + DSS; grade 1.9 ± 0.1) compared to the DSS controls. These mice exhibited reduced inflammatory cell infiltration and fewer pathological changes in the mucosa or epithelial layer. Conversely, histological alterations in the colonic mucosa of GF mice were mild. Control mice treated with DSS displayed increased infiltration of inflammatory cells into the lamina propria and partial disappearance of mucosal crypts in the colonic wall (group DSS; grade 1.7 ± 0.3). Mice pretreated with butyrate for 1 week and cotreated with butyrate and DSS the following week (1BT + BT/DSS group; grade 1.1 ± 0.3) exhibited subtle signs of inflammation. However, mice pretreated with SB for 2 weeks showed a reduced impact on DSS-induced histological changes (2BT + DSS group; grade 1.4 ± 0.2) compared to the DSS controls.

Compared with control animals (CTRL group), we found a significant reduction in crypt length in the DSS group, independent of microbial status, with the length being 2.2 times shorter in SPF mice and 1.5 times shorter in the GF group (*p* < 0.0001; Figure 4B); there was also detected more than 2 times upregulation of *IL-1*β expression in DSS-treated SPF mice compared to control animals (*p* < 0.05; Figure 4C). In SPF mice, the co-administration of butyrate and DSS (1BT+1BT/DSS group) prevented crypt shortening (*p* < 0.0001) and the upregulation of *IL-1*β expression (*p* < 0.05). On the other hand, pretreatment of SPF mice with butyrate before induction of DSS colitis (2BT + DSS group) did not mitigate the impact of DSS on the expression of *IL-1*β, although we observed significant reduction in the effect of DSS on crypt length, compared to mice of DSS group. In GF mice, the administration of butyrate failed to alleviate the DSS-induced reduction in crypt length in both 1BT+1BT/DSS group and 2BT + DSS group, and the expression of *IL-1*β was decreased following the co-administration of butyrate and DSS (1BT+1BT/DSS group) compared to that of untreated, control GF mice (CTRL group) (Figures 4B, C). The patterns of butyrate and DSS effects on *Tnfα* expression were similar but did not reach statistical significance. These data indicated that a serious inflammatory response occurred in the mouse colon and that tissue damage could occur simultaneously. A detailed description of the clinical score and the histopathological changes in SPF mice was already published in our previous publication (Jourova et al., 2022), as well as the clinical score and the length of the colon in GF mice (Satka et al., 2022).

**FIGURE 5:**
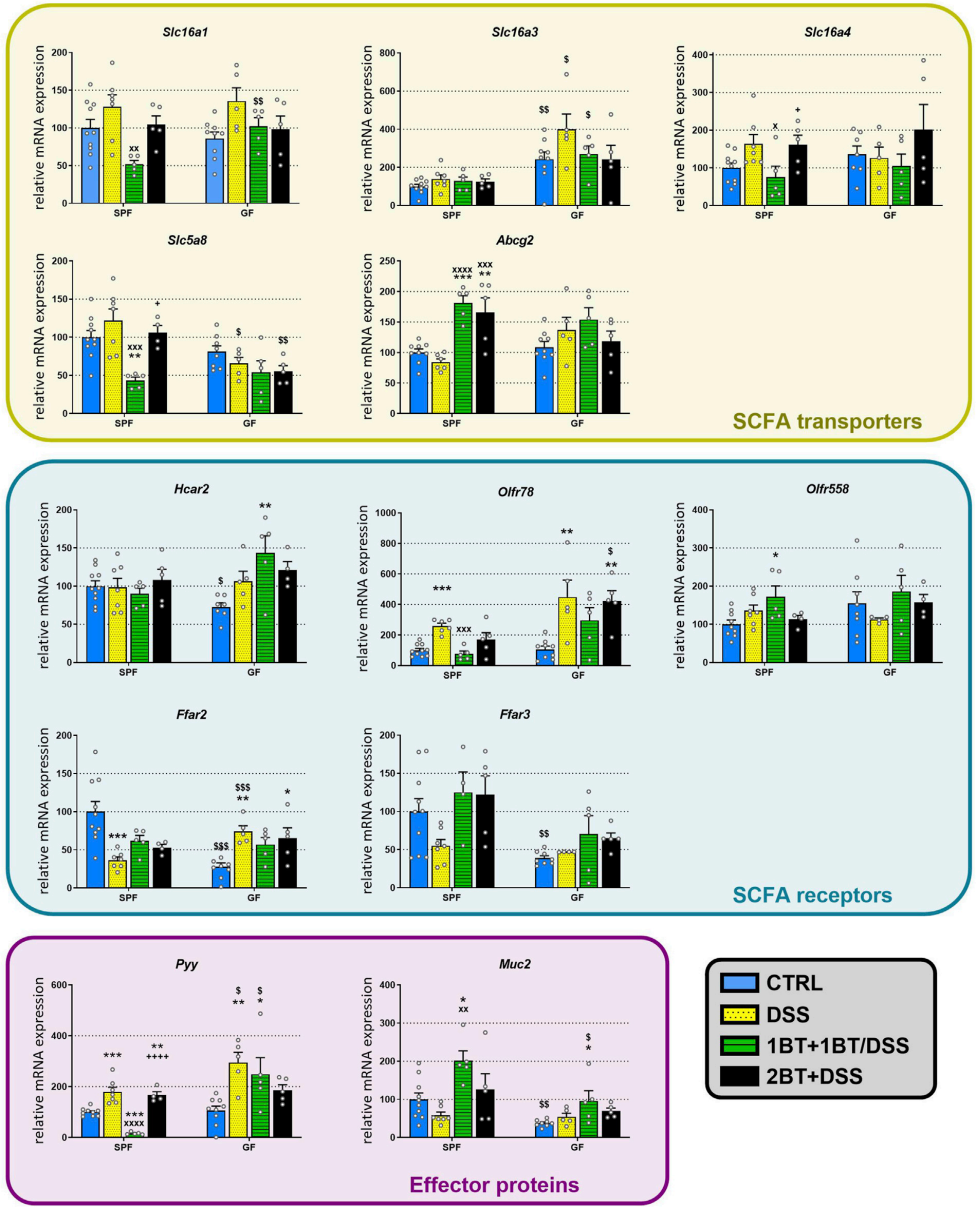
Effect of oral butyrate administration on colonic SCFA transporters and receptors, gut hormone PYY, and mucin 2 in specific pathogen-free (SPF) and germ-free (GF) mice treated with DSS. CTRL, control, untreated mice; DSS, mice that drank 2.5% DSS for 7 days; 1BT+1BT/DSS, mice that drank 0.5% BT solution for 1 week and then the mixture of 0.5% BT and 2.5% DSS for the second week; 2BT + DSS, mice pretreated with 0.5% BT for 2 weeks before drinking 2.5% DSS the following week. The results are presented as the mean ± SEM (*n* = 4–10 per group) and were analyzed by one-way ANOVA separately for SPF and GF mice. Different from the CTRL group: *p < 0.05, **p < 0.01, ***p < 0.001; different from the DSS group: ^x^ *P* < 0.05, ^xx^ *P* < 0.05, ^xxx^ *P* < 0.001, ^xxxx^ *P* < 0.0001; different from the 1BT+1BT/DSS group: ^+^ *p* < 0.05, ^++++^ *p* < 0.0001. Differences between SPF and GF mice in identical experimental groups were compared using Student’s t-test: ^$^ *p* < 0.05, ^$$^ *p* < 0.01, ^$$$^ *p* < 0.001.

Challenge with DSS did not change the mRNA expression of SCFA transporters in the colon regardless of the presence or absence of microbiota (Figure 5). Co-administration of DSS and butyrate (1BT+1BT/DSS group) significantly altered the expression of *Slc16a1* (*p* < 0.01), *Slc16a4* (*p* < 0.05), *Slc5a8* (*p* < 0.001) and *Abcg2* (*p* < 0.0001) in SPF but not GF mice treated with DSS (DSS group). Except for *Abcg2*, DSS had a similar effect on the expression of SCFA transporters if it was applied alone or after a preceding 2-weeks administration of butyrate. The expression of SCFA receptors was only partially affected by DSS. DSS significantly downregulated the expression of *Ffar2* (*p* < 0.001) in SPF mice and upregulated the expression in GF animals (*p* < 0.01); this effect showed a tendency to persist in the case of co-administration or pretreatment with butyrate. In contrast, DSS upregulated *Olfr78* expression in both SPF (*p* < 0.001) and GF animals (*p* < 0.01) and co-administration of DSS and butyrate (1BT+1BT/DSS group) prevented this upregulation in SPF but not GF mice. Like *Olfr78*, the expression of *Pyy* was also upregulated in the presence of DSS in both SPF and GF mice and the co-administration of DSS and butyrate successfully prevented this upregulation in SPF mice but not GF mice.

## Discussion

The mechanism by which SCFAs, particularly butyrate, promote immunity and improve IBD treatment efficacy is not completely understood, even though it is known that SCFAs act at the cell surface as endogenous ligands for some G protein-coupled receptors and intracellularly as inhibitors of histone deacetylases (Gonçalves et al., 2018; Dalile et al., 2019). Previous studies have shown that the administration of sodium butyrate in drinking water ameliorates inflammation and epithelial barrier dysfunction (Vieira et al., 2012; Ji et al., 2016; Lee et al., 2017; Chen et al., 2018; Jourova et al., 2022); nevertheless, orally administered butyrate is thought to be absorbed and utilized before reaching the colon (Daniel et al., 1989; Wang et al., 2023). Here, we demonstrate that a relatively low concentration of sodium butyrate in a drinking solution modulated the expression of the SCFA carriers *Slc16a1*, *Slc16a3*, *Slc16a4,* and *Slc5a8* and the efflux pump *Abcg2*. The observed effect of butyrate on the expression of SCFA transporters does not seem to reflect direct substrate-induced regulation *via* butyrate. First, the effect of oral butyrate differed between SPF and GF mice. Second, we found downregulated expression of SCFA solute carriers in SPF mice treated with butyrate, but butyrate stimulated the expression and function of *Slc16a1* (MCT1) and *Slc16a3* (MCT4) in colonic epithelial cell lines (Borthakur et al., 2008; Ziegler et al., 2016)) or in the intestinal epithelium (Dengler et al., 2015). Third, oral administration of butyrate significantly upregulated *Slc16a3* expression but only in GF and not SPF mice.

In this report, we also showed that not only the expression of SCFA transporters but also that of SCFA receptors is modulated by the gut microbiota and by oral intake of butyrate. We found that the expression of *Ffar2*, which is expressed both in enteroendocrine cells (EEC) and colonocytes, and *Ffar3*, which is expressed in EEC and enteric neurons but not colonocytes (Priyadarshini et al., 2018), strongly depends on gut microbiota but not on oral butyrate treatment. A similar effect of the microbiota on the expression of *Ffar2* was observed in the mouse ileum (Yajima et al., 2016). In contrast to *Ffar2* and *Ffar3*, the expression of *Hcar2*, a colonic SCFA receptor (Priyadarshini et al., 2018), depended not only on gut microbiota, as was already shown earlier by Cresci et al. (Cresci et al., 2010), but also on oral butyrate. Similar to the colonic *Slc16a1* and *Slc5a8* transporters, oral butyrate dampened *Hcar2* expression in SPF but not GF mice. These data indicate a relationship between the regulation of *Hcar2*, the principal butyrate receptor in the colon, and the colonic butyrate transporters *Slc16a1* and *Slc5a8*. A similar link between the expression of *Hcar2* and *Slc5a8* was shown by Cresci et al. (Cresci et al., 2010). The importance of the microbiota for the effect of oral butyrate on the gut is also supported by the results of *Pyy* and *Muc2* expression. In both cases, the effect of butyrate was evident only in SPF but not GF animals, with downregulation in the case of *Pyy* expression and upregulation in the case of *Muc2* expression; this effect is similar to the effect of rectal butyrate enemas on *Muc2* expression in conventional mice (Gaudier et al., 2009). The lack of the inhibitory effect of butyrate on *Pyy* expression observed in our *in vivo* experiments with GF mice aligns with findings from experiments conducted using sterile mouse intestinal epithelial cell culture (Larraufie et al., 2018).

Indirect transcriptional regulation of colonic SCFA transporters and receptors may depend on the interaction of oral butyrate with the gut microbiota or with upstream parts of the gastrointestinal tract. In the case of the effect of oral butyrate in SPF mice, we cannot exclude the possibility that oral butyrate modulates the gut microbiota and microbiota-secreted soluble factors, which might control the expression of SCFA transporters and receptors. There is strong evidence that oral sodium butyrate remarkably alters the gut microbiota (Dou et al., 2020; Lee et al., 2022) and that *Lactobacillus plantarum*-derived soluble factors or *Lactobacillus delbrueckii* consumption upregulate *Slc5a8* and *Slc16a1* expression (Hou et al., 2022; Kim et al., 2022). Furthermore, the SMCT1, MCT1, and MCT4 transporters and the FFAR2 and FFAR3 receptors have been found in the duodenum (Akiba et al., 2015; Kaji et al., 2015), and butyrate has been shown to activate vagal afferents and release gut hormones that might modulate intestinal transport (Dalile et al., 2019).

Since butyrate has ameliorative effects in the treatment of colitis (Recharla et al., 2023), and we have shown that butyrate together with the microbiome affects the expression of transporters and receptors in the colon, we further investigated the interaction between oral treatment of butyrate and the presence/absence of microbiome in a model of acute colitis. Even though downregulation of *Slc16a1* and MCT1 protein expression and upregulation of *Slc16a3* and MCT4 expression were detected in biopsies of patients with IBD and in the colon of rats or mice with DSS-induced colitis (Thibault et al., 2007; Erdmann et al., 2019; Zhang et al., 2019), our results did not show any effect of DSS on SCFA transporters in SPF mice. This discrepancy may result from differences in the colitis models used including strain, time of colitis, and degree of inflammation. In this regard, the expression levels of SCFA transporters depend on the inflammatory state of the colonic mucosa (Thibault et al., 2007; Zhang et al., 2019). Moreover, DSS treatment significantly alters the structure of the intestinal microbiota (Lin et al., 2023), whose diversity and composition might differ among various locations/research groups.

In addition, we analyzed the expression of SCFA receptors in mice with colitis and demonstrated that DSS treatment leads to upregulation of *Olfr78* expression regardless of the presence or absence of gut microbiota and significant downregulation of *Ffar2* expression in SPF mice and upregulation in GF mice. In addition, *Ffar3* expression in SPF mice was also decreased, but the decrease was not significant. In contrast, *Hcar2* and *Olfr558* expression was not changed in either SPF or GF mice. In this regard, acute DSS colitis in conventional C57BL/6 mice decreased the expression of *Ffar2*, *Ffar3,* and *Hcar2* (Lin et al., 2023; Nan et al., 2023), although this result was not confirmed by others (Kotlo et al., 2020; Han et al., 2021). These findings indicate that *Olfr78*, *Ffar2,* and *Ffar3* may play a more important role in our model of colitis/inflammation than other SCFA receptors and that the response of *Ffar* receptors to inflammation depends on the gut microbiota.

In conclusion, our study demonstrates that the intestinal microbiota actively modulates the response of genes encoding colonic SCFA transporters and receptors, mucin, and the gut hormone PYY to oral butyrate administration. It is noteworthy that the responses to butyrate did not differ significantly between the colons of healthy subjects and those with experimentally induced murine colitis. Our observations indicate alterations in the expression of certain SCFA receptors and the hormone PYY in colitis, while the expression of SCFA transporters remained unchanged. It is important to note that these changes occurred regardless of the presence or absence of gut microbiota. Overall, our results suggest a promising avenue for further research in this area. These findings highlight the potential indirect influence of butyrate supplementation on its anti-colitic effects.

## Data Availability

The raw data supporting the conclusion of this article will be made available by the authors, without undue reservation.
